# A Patient With Mental Disorder Wrongly Detained in a European Migrant Detention Centre

**DOI:** 10.7759/cureus.35556

**Published:** 2023-02-27

**Authors:** Gustavo França, Mafalda Corvacho, Catarina Cunha, Barbara Almeida

**Affiliations:** 1 Porto Community Mental Health Service, Centro Hospitalar Universitário de Santo António, Porto, PRT; 2 North Delegation, Doctors of the World, Porto, PRT; 3 Psychiatry Department, Centro Hospitalar Universitário do Algarve, Porto, PRT

**Keywords:** transcultural psychiatry, global mental health, wandering, psychopathology, detention centers, refugees, undocumented migrants

## Abstract

The admission of undocumented migrants and refugees to detention centres (DC) has been systematically associated with several poor mental health outcomes. Much less is known about people with mental health disorders, non-migrants, who might be wrongfully committed to these facilities. This article draws on Dave’s case, where a German citizen was detained in a migrant DC in Porto. The patient was later treated and diagnosed with schizophrenia. In light of another case report, we conceptualize the “Cornelia’s phenomenon” by which a person with full citizenship rights but with a severe mental disorder is wrongly committed to a DC. We hypothesize that this worrisome phenomenon is underestimated, and we will discuss how pre-existent psychopathology might predispose vulnerable people to this situation. We will discuss the negative impact that detention might have on these patients, proposing solutions that might amend this worrisome phenomenon.

## Introduction

The number of asylum seekers, refugees, and internally displaced people worldwide has increased dramatically over the last decades which forced the creation of numerous detention centres (DCs) [1]. The main purpose of these centres is to identify persons and determine nationalities, preventing migrants from gaining unauthorized entry, and expelling or ensuring the enforcement of a deportation order. However, these DCs have raised several doubts regarding their efficacy and ethical uncertainties regarding human rights, freedom of movement, and obligations to non-citizens [2]. Since the beginning, the medical community has raised concerns that detention might exacerbate or trigger the new onset of mental health conditions. There is solid evidence that detention is associated with large negative psychological outcomes [3]. In fact, levels of post-traumatic stress disorder (PTSD), depression, and anxiety were found to be higher among asylum seekers who were detained compared to those who were not detained [4,5]. In most studies, the time in detention has a temporal association with the severity of distress [6]. But, even if the detention is temporary, following release from DCs, many detainees suffer an ongoing sense of insecurity and injustice, difficulties with relationships, and profound changes to their view of self and poor mental health [7]. Theoretically, these negative mental health outcomes are even more pronounced in people with a predisposing vulnerability, such as a history of trauma (as the detention might act as a new stressor, leading to an increased likelihood of developing PTSD or other disorders) [3], or a pre-existent mental illness. Although the corpus of empirical studies outlines the extent and severity of psychopathology observed in DCs, much less is known about mental health patients, non-migrants, who might be illegally committed to these facilities, primarily in virtue of a non-treated mental health disorder. To date, there is a paucity of studies and reports on this subject, and the literature relies on the famous case of Cornelia Rau [8]. We will present Dave’s case, a German citizen who was wrongly detained in a DC, in Porto, in 2020. Then, we will discuss some of the practical implications of this case. The patient provided written informed consent for this publication.

## Case presentation

Dave (fictitious name) was a 39-year-old white person who was seen wandering in a middle town of Portugal, Aveiro. When he was found by the police, he refused to provide his identification, nor did he have any personal documents. He was speaking English with an Eastern European accent. Declining to collaborate with authorities, he was arrested and committed to a migrant DC in Porto (Unidade Habitacional de Santo António - UHSA), which is ruled by Foreigners and Borders Service (Serviço de Estrangeiros e Fronteiras). This DC has the institutional support of the Doctors of the World (DW), a humanitarian organization that runs several projects in Portugal and worldwide. In Porto, DW provides medical assistance to undocumented migrants, asylum seekers, and refugees detained at UHSA.

Dave presented incoherent speech and disorganized behaviour during his first days of admission in the DC. He claimed to be an American citizen, working for the USA Navy, on a “secret mission.” He also stated that he was a victim of experiences on him, and when asked about his biological parents or any relatives, he said he was conceived in a laboratory. Following two observations by our psychiatry team, he was admitted to a psychiatry inpatient facility (Hospital de Magalhães Lemos) on 13th August 2020 with a provisional diagnosis of psychosis. Dave wandered from Kiel (northern Germany) to Porto, which is a journey of 2,566 km. He had a history of hospitalizations in Germany.

During the first days at the hospital, Dave was very defensive, suspicious, and unable to collaborate, presenting a polymorphous delusion with persecutory elements and grandiosity. He did not show signs of significant cognitive deterioration. He received treatment with risperidone (which was increased up to 10 mg per day). During the treatment, he became more collaborative, and his psychotic symptoms showed partial remission following the treatment.

On 29th December 2020, he was discharged on risperidone 6 mg per day and lorazepam 1 mg, with a final diagnosis of schizophrenia (F20, ICD-10) - paranoid subtype. Thanks to articulation with German Embassy, we were able to confirm his identity and provide a new passport that made possible his flight back to Germany on the same day.

## Discussion

The discussion will proceed threefold. First, we will discuss why psychopathology might predispose to the “Cornelia’s phenomenon,” by which a person with full citizenship rights but with a mental disorder is wrongly committed to a DC. Then, we suggest that schizophrenia, in particular, might predispose to this phenomenon, based on its phenomenological psychopathology and recent findings of evolutionary psychology. Finally, we will discuss the impact that detention might have on these patients and propose solutions that might amend this worrisome phenomenon.

Cornelia’s phenomenon to occur requires four conditions. First, the “unidentified” patient must have the psychomotor ability to move autonomously and eventually cross borders. Second, the patient must have a psychopathological condition severe enough to interfere with the subjective experience of his identity. Third, the patient must show disorganized behaviour or not take care of himself in a strange environment, to the point of drawing the attention of others. Finally, the psychopathological picture cannot be completely obvious to the authorities so he is admitted to a DC instead of being admitted directly into a hospital unit.

Fortunately, most people with mental illness have the significant capacity to walk and the freedom to move, including journeys to different regions and countries (this is particularly relevant in community spaces, such as the European Schengen area). This freedom is just compromised in cases of acute admission or when the patient is institutionalized (where some rules and limitations might apply).

From a theoretical point of view, several psychopathological syndromes can lead to a disturbance of identity. One of the most common in clinical practice is impaired cognitive functions, namely neurodegenerative disorders, in which the patient might show disorientation for space and time and disorientation toward himself. He might also show delusions of identity or confabulations regarding his biography [9]. Effectively, the risk of patients with neurodegenerative disorders getting lost is significant. This burden requires frequent intervention by caregivers and sometimes leads to institutionalization. Another potential situation, albeit more unusual, is the dissociative syndromes. Dissociative identity disorder, dissociative amnesia, depersonalization, and derealization are core phenomena of dissociative psychopathology. Dissociation is defined as a disruption and/or discontinuity of the normal subjective integration of behaviour, memory, identity, consciousness, emotion, perception, body representation, and motor control. A dissociative fugue is characterized by amnesia and a sudden unexpected travel away from the individual’s usual surroundings and denial of all memory of his whereabouts during the period of wandering. These patients might temporarily lose their identity and wander across space, getting lost and ending up in emergency wards. These episodes are usually brief; however, in some cases, the length of the fugue may last several months. Finally, a psychotic episode, in the context of a mood or primary psychotic disorder, might also lead to this phenomenon. This was the case with Cornelia Rau and Dave (our patient) so that we will discuss it in more detail.

Schizophrenia is a chronic, heterogeneous behavioural and cognitive syndrome that seems to originate from disruption of brain development caused by genetic or environmental factors, or both [10]. It is expressed as a combination of psychotic symptoms (delusions, hallucinations, and disorganized behaviour), negative symptoms, and cognitive dysfunction. Moving beyond the third-person perspective of schizophrenia, a phenomenological perspective, integrating a first-person account of schizophrenia is essential to grasp these patients' lived experiences and complex behaviour. Some authors have proposed an ipseity-disturbance or self-disorder hypothesis regarding schizophrenia [11], emphasizing anomalies of self-awareness (the fading first-person perspective, the waning sense of basic identity, and depersonalization), the loss of common sense, and existential alterations [12]. The patient feels ephemeral, lacking a core identity, and profoundly different from others and alienated from the social world. In an extreme condition, the patient might lose the capacity to tell his name or to remember his past. The multidomain cognitive deficits might manifest as delusional memories and confabulation, hindering, even more, the development of a stable narrative self. Contributing to this fragility, schizophrenic patients may also show a delusional orientation (or the phenomenon of “double bookkeeping”) and age disorientation, which might be an indicator of severe psychopathology [13].

Mainly in an acute phase of the disorder, schizophrenia is usually accompanied by delusions that can directly contribute to Cornelia’s phenomenon. An example is a patient with a persecutory delusion who might believe to be persecuted by his fellows and so runs away from his country. Another example is a patient with a delusion of grandeur who might believe he has a mission to evangelize people from other countries. In the context of a radical critique of psychoanalysis, Deleuze and Guattari famously wrote in Anti-Oedipus (1983) “A schizophrenic out for a walk is a better model than a neurotic lying on the analyst's couch.” For these authors, the nomadic and disruptive “schizophrenic” was a model to follow, as the producer of a desire and a divergent thought that lies outside and beyond the existing frames of reference [14]. Effectively, the nomadic and somehow erratic behaviour is also characteristic of some patients with schizophrenia. Schizophrenia is the medical diagnosis most often associated with the risk of absconding behaviour, in medical facilities [15]. Wanderers were more likely to have symptoms of delirium, exhibit socially inappropriate behaviour, manifest problems in decision-making, and to take anti-psychotic medication [16]. Interestingly, airport wandering has been proposed as a psychotic symptom [17]. Clinical practice shows that many patients live a nomadic lifestyle, even within a limited community, entering an “institutional circuit” where they may transit among institutions (hospitals, prisons; nursing homes; streets). In this context, temporary shelters might be a fundamental resource in essentially nomadic lives [18].

Finally, an evolutionary perspective can help to understand this wandering phenomenon. Schizophrenia is associated with a broad spectrum of genes that make the brain vulnerable or less adaptive to the environment. Evolutionary theory may be relevant to schizophrenia, and various evolutionary theories have speculated on the origins of schizophrenia. Stevens and Price have put forward a “group-splitting hypothesis of schizophrenia” in which they assume that proliferating tribal communities must eventually split [19]. Psychotic symptoms might be adaptive mechanisms in a specific environmental context. For example, being hyper-alert and “paranoid” could be useful to interpret signs and details in a different surrounding and to infer the mental state of others and their intentions. Accordingly, most delusional beliefs have a persecutory nature. The psychosis could be a “natural” adaptative mechanism when one goes from one village to another to start his own life. Thus, from an evolutionary perspective, the psychological mechanisms that detect, anticipate, and avoid social threats can be favoured.

Bearing in mind the paucity of data, we hypothesize that the Cornelia's phenomenon is underestimated. Nevertheless, several preventive measures could help to diminish the risk of wrongly admitting patients with mental disorders in DCs. Most patients with severe mental illness, such as schizophrenia, are treated by community teams and have some institutional support in their daily living activities. In some daycare facilities, patients may be deprived of their personal identification documents (sometimes with justified fears of loss); hence, the use of identification wristbands might be valuable. Another measure could be creating an international database of missing persons with mental disorders, provided the issues of confidentiality of data are considered. A medical screening, including a basic neuropsychological assessment, is also of paramount importance to every detained, before admission in any DC. This is contemplated in most guidelines and protocols involving migrants, but it is not always applied in practice. Finally, the authorities that deal with this group must have basic skills in mental health, so they can identify situations that benefit from a specialized medical evaluation. These skills can be integrated into the training of every clinical and staff working in DC.

As was said before, admission into a DC can have a devastating effect on people’s mental health and well-being. This effect is amplified when we are dealing with people with pre-existing mental disorders. The DC is not an appropriate place to accommodate people with mental disorders. There should be alternatives to detention in the case of mental health patients, such as community accommodation, which is more human and can lead to better health outcomes. In addition to unnecessarily subjecting a person to a closed, freedom-restrictive and potentially violent environment, there is a potential delay in medical diagnosis and treatment, which can have quite negative consequences.

Doctors of The World (Médicos do Mundo) and other humanitarian associations struggle to improve the delivery of mental health care within the context of immigration detention. This is a very well-known and identified gap. During the last years, in Porto, there was a need to transfer several patients to the psychiatry hospital for a higher level of treatment [20]. One of the main difficulties is the continuity of care. Our intervention is targeted at the acute crisis. Still, only the longitudinal view of psychiatry treatment might target the social determinants of the post-migration context, focusing on recovery and relapse prevention. Another difficulty is the lack of a multidisciplinary team and the lack of psychosocial treatments. 

## Conclusions

This case report points out that several patients with serious mental illness may be unduly detained in DCs for migrants. The psychological impact of detention can further aggravate their psychopathological state. Patients like Dave are left (and maybe forgotten) in a “paradoxical limbo.” On the one hand, they are detained and isolated from their family and community. Hence, they can not exit the facility because they do not have documents or deny to provide a valid identity. On the other hand, they can not provide identity because they are mentally unstable. Having defined Cornelia's phenomenon, by which a patient with a severe mental disorder is wrongfully committed to a DC for migrants, we hypothesize that the prevalence of this phenomenon is underestimated. These patients need specialized medical care and treatment, but as they are in a secluded space, which aggravates their mental health, access to specialized healthcare becomes difficult. In Dave's case, the intervention of psychiatry proved to be decisive for his clinical treatment and repatriation to his country of origin. The creation and development of multidisciplinary mental health teams that can provide care to people who find themselves in DC continue to be of vital importance.
